# Mineralogical compositions of soils under three geological formations in some parts of Ogun state, Nigeria and their agricultural potentials

**DOI:** 10.1038/s41598-024-57397-0

**Published:** 2024-03-22

**Authors:** Aruna Olasekan Adekiya, Grace Atinuke Ajayi, Kehinde Abodunde Adegbite, Faith Luciana Imhanze, Ayibanoa Lekoo Ibaba

**Affiliations:** 1 Agriculture Programme, College of Agriculture, Engineering and Science, Bowen University, Iwo, Nigeria; 2 Life on Land Research Group, College of Agricultural Sciences, Landmark University, PMB 1001, Omu-Aran, Kwara State Nigeria; 3 School of Natural and Environmental Science, Newcastle University, Newcastle, UK

**Keywords:** Basement complex rock, Coastal plain sands, Ewekoro formation, Mineralogical composition, Kaolinite, Geology, Mineralogy

## Abstract

Investigating the mineralogical compositions of soils under different geological formations becomes imperative for maximizing agricultural productivity and ensuring the long-term viability of agricultural practices. Therefore, studies were carried out on mineral compositions and diversities of soils developed over the Basement complex rock, Coastal plain sands and Ewekoro formations in Ogun state Nigeria. A total of nine profile pits (three per location) of 2 m × 1 m × 2 m size were dug in all the three locations. Soil samples were collected from the pedogenic horizons of each profile pits in replicates into a well labeled polyethylene bag. Using X-ray diffractometry (XRD) and scanning electron microscopy (SEM) the mineral contents and their relative abundance, elemental compositions and morphologies of the fine sand, coarse silt and clay fractions of the soils at different topographic positions were identified, described and compared. Results obtained from XRD and SEM analyses exhibited similarities. The most abundant elements in the basement complex and Ewekoro pedon were oxygen, carbon and silicon whereas in the coastal plain sand pedon, oxygen, carbon and aluminum were the most abundant element. The presence of mixed-layer illite, mica, kaolinite, quartz, hematite, anatase, goethite, and chlorite at varying degrees was observed in the pedons developed on these geological formations, although kaolinite and quartz dominated the soil matrix. The mineralogical complexity of the pedons followed the order of basement complex > coastal plain sand > Ewekoro formation. Profiles developed on the Ewekoro formation exhibited the highest degree of weathering, as evidenced by their chemical properties and mineralogical compositions. The petrographic evaluation of the three geological formations revealed that all pedons were rich in quartz and exhibited varying degrees of mineral complexity and maturation. The overlapping and distinct characteristics among the geologies indicated different stages of weathering. By using the mineral maturity index, profiles developed over the basement complex rock and the coastal plain sand could be regarded as sub-matured and this could have contributed significantly to the native fertility of these soils and profiles from the Ewekoro formation were the most weathered. The use of Ewekoro formation for agriculture would necessitate significant investments in agro-inputs and sound principles of soil management through integrated soil fertility management.

## Introduction

Soil is a dynamic natural body that is composed of mineral particles, organic matter, water, and air. It is essential for plant growth and plays a vital role in the environment. Soil composition and its mineralogical content play a crucial role in determining the agricultural potentials of a given region. Understanding the mineralogical compositions of soils is essential for effective land management, crop productivity, and sustainable agricultural practices^1,2^. The geological formations underlying a particular area greatly influence the mineralogy of the soils found there. The clay mineralogy and environmental conditions during soil formation establish the inherent physico-chemical characteristics of diverse soils^3^. To comprehend and elucidate any physical or chemical property of soil, it is imperative to understand the composition of minerals like kaolinite, montmorillonite, illite, and various amorphous minerals, in addition to the content of organic matter^4^. Thus, investigating the mineralogical compositions of soils under different geological formations becomes imperative for maximizing agricultural productivity and ensuring the long-term viability of agricultural practices. Clay minerals, through their physical and chemical properties, affect soil fertility by controlling nutrient supplies and availability, through the sequestration and stabilization of soil organic matter, by controlling soil physical properties through microaggregate formation, by influencing soil acidity and controlling soil microbial population and activity. The main processes involved in these relationships are dissolution–precipitation and adsorption–desorption processes, alongside mechanisms involving the formation of short-range-ordered phases^5^. Furthermore, these studies serve the purpose of investigating the genesis of soils and predicting their viability for both agricultural and non-agricultural purposes. It is widely acknowledged that the composition of parent material plays a pivotal role in shaping the formation and attributes of soils, as highlighted by Schaetzl and Thompson^6^.

Ogun state, located in southwestern Nigeria, exhibits a diverse geological landscape, characterized by various rock formations and soil types. The state’s soils are derived from three rock types: sedimentary, metamorphic, and igneous. Each of these geological formations possesses unique mineralogical characteristics, which may significantly influence the soil’s physical, chemical, and biological properties. The diverse soil diversity in this region has long facilitated food production, catering to both peasant farmers and commercial operations. Unfortunately, these soils have not been adequately researched, depriving users of essential data necessary for enhancing their management.

The basement complex rocks in Nigeria constitute a collection of crystalline igneous and metamorphic rocks that originated from the Precambrian to the lower Proterozoic period. Tukur^7^ categorized these rocks into three main lithological units: the migmatite–gneiss–quartzite complex, the schist belt, and the older granites. From a geological perspective, coastal plain sand represents a sedimentary basin situated within the coastal plain sands of the Benin formation, subsequently overlain by alluvial deposits. The stratigraphy of the eastern Benin Basin has been extensively examined by various researchers, leading to the proposal of several classification schemes^8–10^. The Ewekoro formation (Paleocene) stands as one of the stratigraphic units in the eastern Dahomey Basin. It primarily consists of limestone, accompanied by subordinate thin bands of shale, marl, and sand^8^. Unfortunately, these soils have not been adequately researched, depriving users of essential data necessary for enhancing their management. Soil mineralogical study is not a common exercise in the in Nigeria in recent times probably due to the cost of analyses and nonavailability of up-to-date analytical instruments. Clays are best studied and identified using the X-ray diffractometry (XRD) and scanning electron microscopy (SEM)^11^. Few studies^12–15^ have reported the use of XRD and SEM in soil mineralogical study in Nigeria.

The experiment conducted by Ajiboye et al.^12^ explored the influence of soil mineralogical characteristics on sustainable soil fertility management in certain tropical Alfisols in Nigeria. The findings indicated that approximately 75% of the exchangeable bases were attributed to calcium and magnesium, which dominated the exchange sites. In the clay fractions, illite/mica and kaolinite emerged as the primary minerals, whereas quartz, mica, and feldspars prevailed in the fine sand and silt fractions of the soils. The presence of illite and mica was deemed significant for potassium nutrition, while kaolinite and iron oxides were identified as potential contributors to phosphorus fixation. Another study by Aliu et al.^16^ focused on the geochemical composition and mineralogical characteristics of clay soil samples from Afo-Okpella and Okpekpe in parts of the southern Niger Delta in Nigeria. X-ray fluorescence (XRF) analysis revealed that SiO_2_ and Al_2_O_3_ were the predominant oxides. X-ray diffraction (XRD) analysis further indicated that kaolinite was the primary clay mineral, accompanied by varying amounts of quartz and traces of illite and smectite. In southeastern Nigeria, Igwe et al.^17^ conducted a study revealing that the mineralogy of the soils was predominantly characterized by kaolinite. Additionally, smectite observed in certain wetland soils was assumed to have been transported from drier areas.

Understanding these mineralogical compositions is vital for devising appropriate soil management strategies, selecting suitable crop varieties, and optimizing fertilizer application for sustainable agricultural development in the region.

It will contribute to the existing body of knowledge concerning the relationships between geological formations, soil mineralogy, and agricultural potentials in similar regions. The information generated from this research will aid in formulating soil management strategies, land use planning, and crop selection that can maximize agricultural productivity and sustainability in Ogun State, Nigeria.

**Figure 1:**
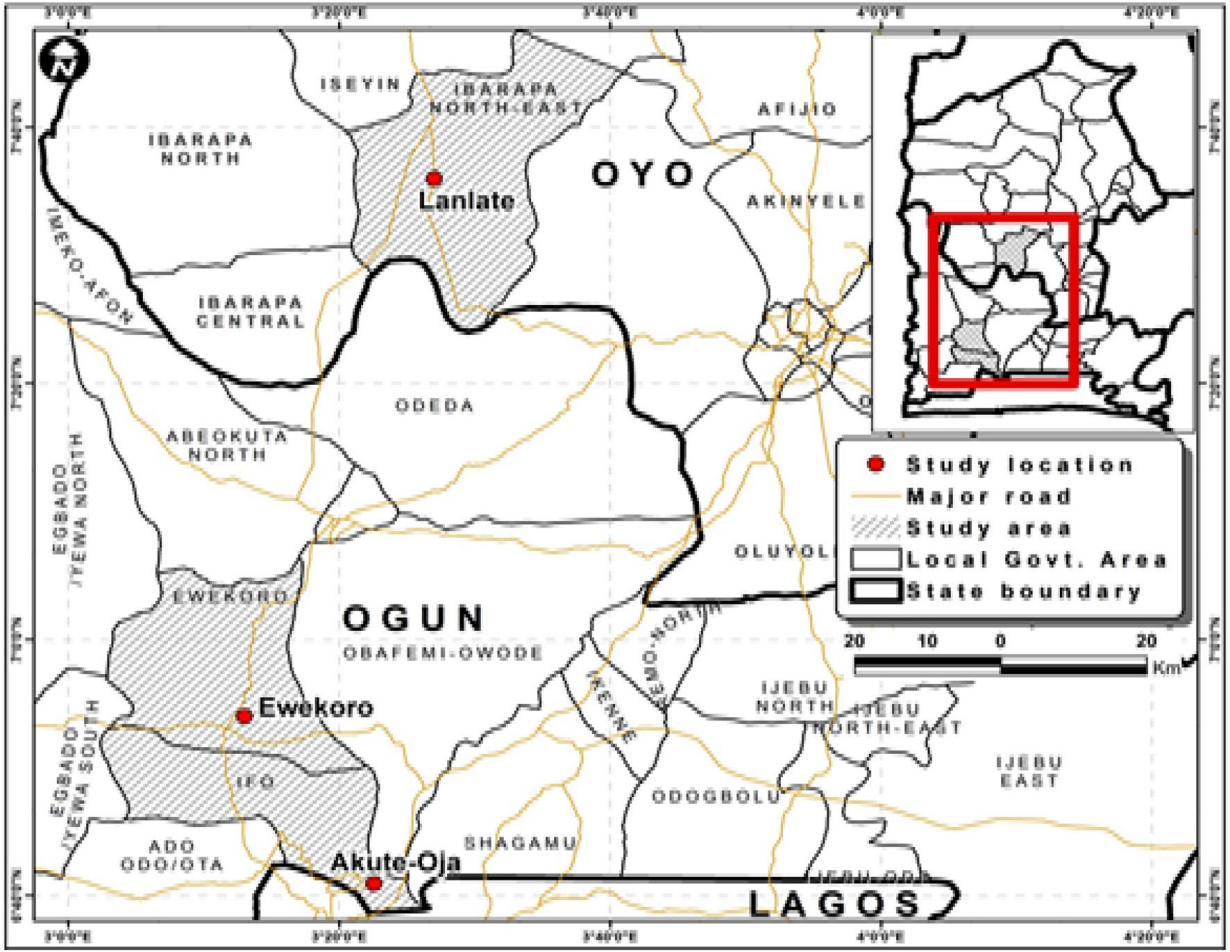
Map of Nigeria showing the three (3) study locations. *Mg* magnesium saturated and glycerol solvated, *K550* potassium saturated and glycerol solvated heated to 550 °C.

Overall, this study emphasizes the importance of understanding the mineralogical compositions of soils under different geological formations and their implications for agricultural productivity. By unraveling the relationships between soil mineralogy and agricultural potentials, this research aims to provide valuable insights that can guide sustainable agricultural practices and promote food security. The objectives of this study are to (i) determine the mineralogical composition of the clay fraction of pedons on different geologies and their implications for agricultural productivity. (ii) Evaluate the mineralogical diversity of fine sand and coarse silt fractions of pedons developed over different geological formations and their implications for agricultural productivity. Based on these objectives, it was hypothesized that there will be differences in the mineralogical composition of clay fraction, fine sand and coarse silt fraction of pedons over different geologies. Experiments were conducted to evaluate these working hypotheses; How does the mineralogical composition of soils, specifically in the clay fraction and fine sand/coarse silt fractions, vary across different geological formations, and what are the implications of these variations for agricultural productivity? (Fig. 1).

## Results

### The mineralogical compositions of the clay fraction of soils developed over different geological formations by X-ray diffractometry

#### Coastal plain sand

**Figure 2:**
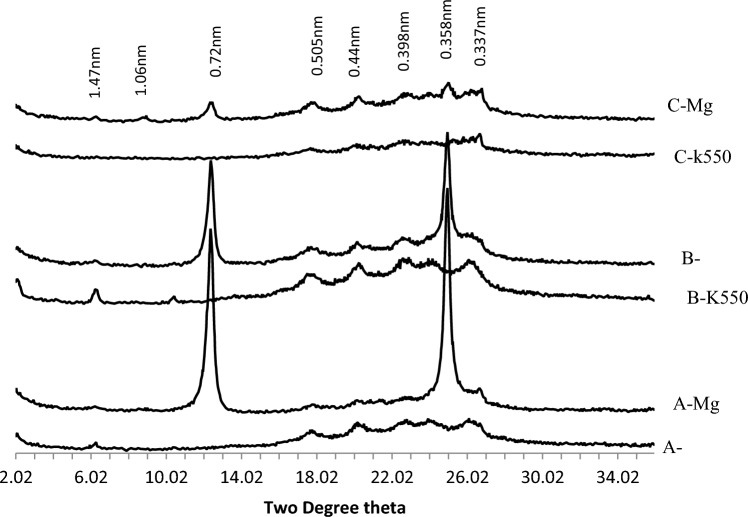
The XRD patterns of the clay fraction of the A, B and C horizons of soil on coastal plain sand formation.

**Figure 3:**
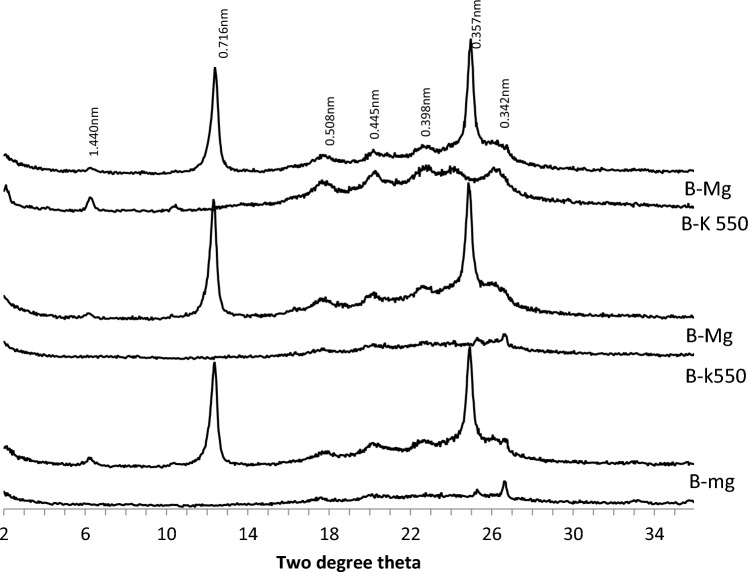
The XRD patterns of the clay fraction of the B horizons of soils on coastal plain sand formation.

XRD patterns of clay fraction in Coastal plain sand soils (Fig. 2) revealed the presence of mixed-layer illite, mica, kaolinite, quartz, hematite, goethite, and chlorite. Kaolinite was confirmed through X-ray patterns in magnesium saturated and glycerol solvated samples, with peaks at 0.716–0.717 nm and 0.357–0.358 nm. These peaks disappeared in potassium saturated and glycerol solvated samples heated to 550 °C. Quartz was indicated by peaks at 0.425, 0.426, 0.335, and 0.339 nm in both magnesium and potassium saturated and glycerol solvated samples. XRD patterns of the B-horizons of the three pedons in coastal plain sand showed the presence of quartz (0.426 nm), kaolinite (0.716, 0.357 nm), degraded mica (0.505 nm), and poorly crystalline goethite (0.45 nm) (Fig. 3).

Table 1 showed the relative abundance of identified minerals in soil phases. Kaolinite and illite were the most abundant, while quartz, hematite, goethite, chlorite, and K-feldspar occurred in minor and trace amounts in the A horizon. In the B horizon, kaolinite, quartz, and illite were present at moderate levels, while hematite and K-feldspar occurred in minor quantities. Chlorite was present at trace levels, and goethite was not detected. In the C horizon, quartz, illite, and K-feldspar occurred in minor quantities, while kaolinite and goethite were found in trace amounts.

**Table 1:** Relative abundance of the identified minerals phase in the soils.

Location	Horizon	Kaolinite	Quartz	Illite/Mica	Hematite	Goethite	Chlorite	K-feldspar
Coastal plain sand	A	++++	+	++++	+	++	++	+
	B	+++	+++	+++	++	nd	+	++
	C	+	++	++	Nd	+	Nd	++
Basement complex	A	++	++	+	+	nd	++	Nd
	B	++	+++	++++	+	nd	Nd	Nd
	C	+++	++++	+++	++	++	+	+++
Ewekoro formation	A	+++	++	++	Nd	+	Nd	+
	B	++++	++++	+++	Nd	++	+++	++
	C	++++	+++	++	Nd	+++	+++	+++

*nd* not detected.

Mineral symbols—++++  = Abundant (> 30%), +++  = Moderate = (10–30%), ++  = Minor (1–10%), +  = Trace (< 1%).

Table 2 provided quantitative clay mineral contents in selected profiles and horizons using XRD in the magnesium saturated samples. Kaolinite dominated the soil matrix, ranging from 60 to 93%. Pedon 01, at a depth of 55–82 cm, had a significant amount of illite/mica at approximately 23%, while other minerals were present in trace amounts, typically less than 8%.

**Table 2:** Quantitative clay mineral content in the magnesium saturated samples.

Geology	Pedon	Horizon depth (cm)	*R0 M-L I/S 90S	Illite and Mica	Kaolinite	Chlorite	Quartz	Anatase	Hematite	Total
					A^0^					
Basement complex	1	0–26	1	6.8	83.2	5.3	3.7	0	0	100
	1	44–65	0	1.1	89.2	2.3	1.5	1.9	4	100
	1	26–44	0	2.2	91.9	4.4	1.5	0	0	100
	2	5–33	0.5	2.4	90.8	2.3	1.4	0.9	1.7	100
	3	12–50	0.7	7.4	77.1	2.2	9	0	3.6	100
Coastal plain sand	1	55–82	0	22.9	59.9	8.4	8.8	0	0	100
	1	40–55	0	1.3	93.4	1.6	1.1	0	2.6	100
	1	0–40	0	1.4	93.1	2.4	1.2	0	1.9	100
	2	35–74	0	0.8	93.3	2.8	0.9	0	2.2	100
	3	23–71	0	0.6	93.4	1.4	1.5	0	3.1	100
Ewekoro formation	1	18–38	0	1.6	78.8	2.7	16.9	0	0	100
	1	38–69	0	0	67.6	15.2	17.2	0	0	100
	1	0–18	0	3	86.8	3.4	6.8	0	0	100
	2	18–35	7.5	0	80.8	5.4	6.3	0	0	100
	3	23–68	0.4	1.4	85.4	6.5	4	2.3	0	100

*R0 M-L I/S 90S—R0 Ordered Mixed-Layer Illite/Smectite with 90% Smectite layers, A^0^ = Amstron.

#### Basement complex rock

**Figure 4:**
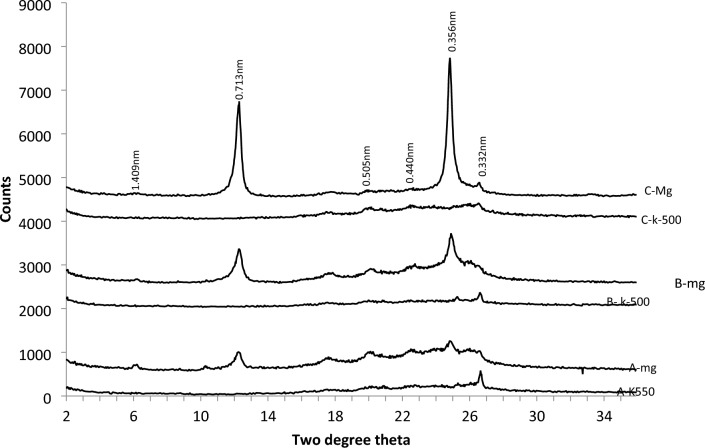
The XRD patterns of the clay fraction of the A, B and C horizons of soil on basement complex rock.

**Figure 5:**
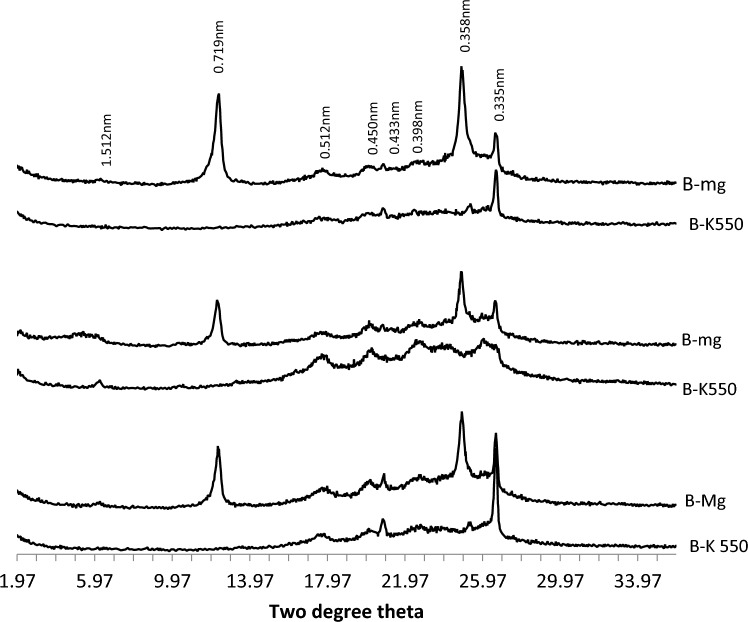
The XRD patterns of the clay fraction of the B horizons of soil on basement complex rock. *Mg* magnesium saturated and glycerol solvated, *K550* potassium saturated and glycerol solvated heated to 550 °C.

The XRD analysis revealed the presence of several minerals in the A, B, and C horizons of pedons developed on the basement complex rock (Fig. 4). These minerals include mixed smectite/mica (1.83 nm), kaolinite (0.72 nm, 0.36 nm), quartz (0.43 nm), degraded mica, and chlorite (0.354 nm). The degraded mica found in the B horizons is likely derived from illite-mica (0.334 nm). The presence of a broad and irregular peak (0.359 nm) suggests the existence of mixed layers containing expandable layers. Additionally, gibbsite was identified in the B horizon (Fig. 5). The mineralogical compositions of the B horizons of the three pedons on the basement complex rock showed considerable similarities.

Table 1 provides information on the relative abundance of the identified minerals in the soil phase. In the A horizon, kaolinite, quartz, and chlorite were present in minor quantities, while illite and hematite occurred in trace amounts. The B horizon exhibited an abundance of illite/mica and a moderate level of quartz. Hematite was found in trace amounts. The C horizon of soils developed over basement complex rock contained abundant quartz, moderate levels of kaolinite, illite, and K-feldspar, and minor or trace quantities of hematite, goethite, and chlorite.

Quantitative clay mineral analysis using XRD on selected pedons and horizons in the magnesium saturated clay fractions revealed that kaolinite dominated the soil matrix, ranging from 77 to 92% in percentage (Table 2). The proportion of illite/mica varied from 1.1% in pedon 01, at a depth of 44–65 cm, to 7.4% in pedon 03, at a depth of 12–50 cm. Chlorite and other minerals were present in trace amounts, typically less than 5%.

#### Ewekoro formation

**Figure 6:**
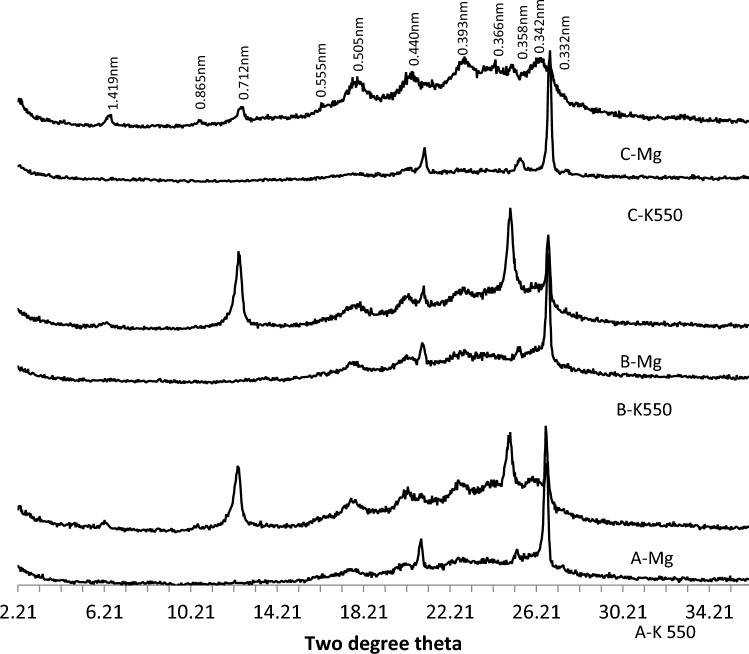
The XRD patterns of the clay fraction of the A, B and C horizons of soil on Ewekoro formation.

**Figure 7:**
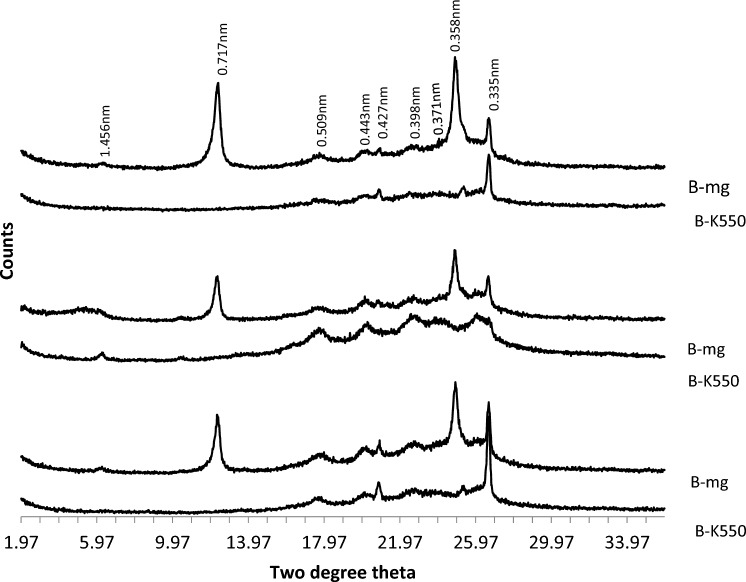
The XRD patterns of the clay fraction of the B horizons of soils on Ewekoro formation.

The clay fraction of pedon developed on the Ewekoro formation contained kaolinite and quartz as the dominant minerals and has variable quantities of illite/mica, goethite, chlorite and K-feldspar in the A, B and C horizons (Fig. 6). Hematite was not detected. The B and C horizons of the pedon were mineralogically more complex than the A horizon. There was no mineralogical diversity between the B horizons of the pedons (Fig. 7).

In term of relative abundance of the identified minerals in soil phase (Table 1), only kaolinite occurred at moderate level other minerals such as quartz, illite/mica, and goethite were either in minor quantities or not detectable on the A horizon. In B horizon, kaolinite and quartz were the most abundant while illite and chlorite occurred at moderate levels. On the C horizon of soils developed over the Ewekoro formation, kaolinite was abundant, quartz, goethite, chlorite and K-feldspar were found at moderate levels. In the magnesium saturated clay fractions of the pedons developed on Ewekoro formation, kaolinite dominated the soil matrix with percentages ranging from 68 to 87% with significant quantities of quartz in the B horizon of pedon 01 (Table 2). Illite/mica occurred in trace amounts in all the pedons. Chlorite and other minerals occurred in trace amounts with the exception of pedon 01, 38–69 cm which had 15% chlorite.

### The mineralogical diversity of the clay fraction of pedons developed over different geological formations using scanning electron microscopy (SEM)

The elemental composition of clay fraction from soils developed over three different geological formations using scanning electron microscopy (SEM) showed that five elements abound in the clay fraction, which were carbon, oxygen, silicon, aluminum and iron at varying amounts (Table 3). A representative pedon was selected for each geological formation. The elemental compoitions of the three geoloigcal formations are as follows.

**Table 3:** Elemental composition of clay fraction by energy dispersion SEM from pedons developed over different geological formations.

Geology	Depth (cm)	O (%)	C (%)	Si (%)	Al (%)	Fe (%)
Basement complex	0–26	63.6	12.2	11.4	8	4.7
	26–44	66.8	6.3	11.7	11.6	3.6
	44–65	63.6	20.1	5.9	6.5	3.9
Coastal plain sand	0–40	63.8	18.5	6.6	7.8	3.2
	40–55	63.5	19.3	6.7	7.3	3.1
	55–82	53.6	38.7	5.3	4.4	3.3
Ewekoro formation	0–18	57.8	30.8	7	3.1	1.4
	18–38	62.8	2.3	18.6	11.2	5.1
	38–69	65.8	3.6	16.6	10	4

**Plate 1:**
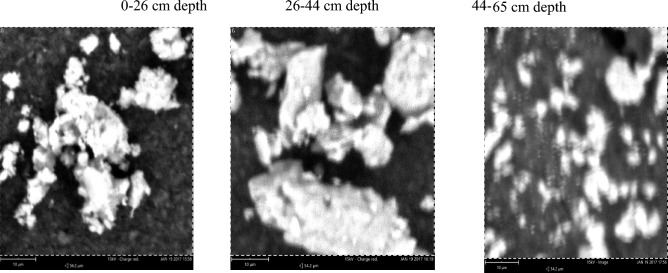
Scanning electron micrographs of soil samples from basement complex rock.

The content of oxygen in soils of the basement complex ranged from 63.6% in the 0–26 and 44–65 cm depth to 66.8% in the 26–44 cm depth whereas carbon varied between 6.3% in the later depth to 20.1% in the 44–65 cm depth. Following the same trend silicon varied between 5.9 and 11.7% and aluminum varied from 6.5 to 11.6% while iron ranged from 3.6% in the 26–44 cm depth to 4.7% in the 0–26 cm depth of the basement complex pedon. The most abundant elements in the basement complex pedon were oxygen, carbon and silicon. The Scanning electron micrographs of the pedon are presented in Plate 1.

**Plate 2:**
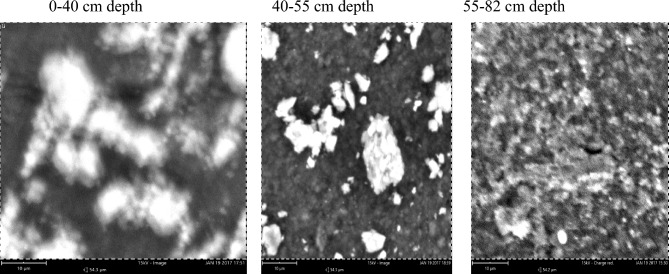
Scanning electron micrographs of soil developed over Coastal plain sand.

On the coastal plain sand, the content of oxygen and aluminum soil varied from 53.6 and 4.4% in the 55–82 cm depth to 63.8% and 7.8% in the 0–40 cm depth (Table 3). Carbon varied between 18.5% at 0–40 cm depth and 38.7% at the 55–82 cm depth. The composition of silicon varied from 5.3 to 6.6% and that of iron was from 3.1 to 3.3%. The most abundant elements in the coastal plain sand pedon were oxygen, carbon and aluminum. The scanning electron micrographs of the pedon are presented in Plate 2.

**Plate 3:**
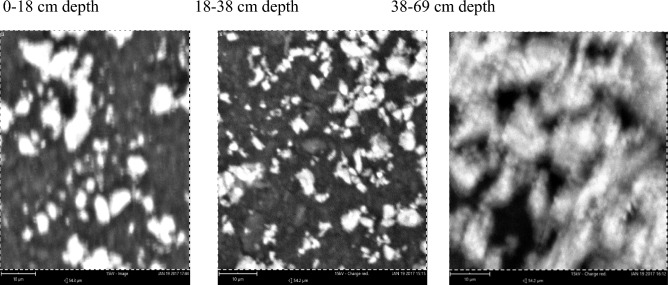
Scanning electron micrographs of soil developed over Ewekoro formation.

Ewekoro formation has oxgyen composition that varied from 57.8% in the 0–18 cm depth to 65.8% in the 38–69 cm depth. The SEM images of the pedon are presented in Plate 3. Carbon varied from 2.3% in the 18–38 cm depth to 30.8% in the 0–18 cm depth. Silicon varied from 7 to 18.6% while aluminum varied from 3.1 to 11.2% (Table 3). Percentage iron composition ranged from 1.4 to 5.1% in the 0–18 cm and 18,038 cm depth, respectively. The the most abundant elements in the pedon of Ewekoro formation were oxygen, carbon and silicon. The scanning electron micrographs of the pedon are presented in Plate 3.

Comparatively, the pedon of basement complex 26–44 cm soil depth had the highest quantity of oxygen with a percentage mass of 66.8% while coastal plain sand 55–82 cm depth had the lowest with a percentage mass of 53.6%. Pedon from coastal plain sand 55–82 cm depth had the highest quantity of carbon with a percentage mass of 38.7% followed by Ewekoro formation 0–18 cm depth which had a percentage mass of 30.8% while Ewekoro formation 18–38 cm depth recorded the lowest amount of carbon. The latter, however, had the highest amount of Si with percentage mass of 18.6% while sample from coastal plain sand 55–82 cm depth had the lowest quantity of Si. The basement complex 26–44 cm depth had the highest amount of aluminum (11.6%) followed by Ewekoro formation 18–38 cm depth which had a percentage mass of 11.2% while Ewekoro formation 0–18 cm depth had the least. Sample from Ewekoro formation 18–38 cm depth had the highest amount of Fe and the least occurred in coastal plain sand pedon at 40–55 cm depth (Plate 3). Carbon varied from 2.3% in the 18–38 cm depth to 30.8% in the 0–18 cm depth. Silicon varied from 7 to 18.6% while aluminum varied from 3.1 to 11.2% (Table 3). Percentage iron composition ranged from 1.4 to 5.1% in the 0–18 cm and 18,038 cm depth, respectively. The most abundant elements in the pedon of Ewekoro formation were oxygen, carbon and silicon.

### The petrological study of soil separates of the formations

#### Fine sand fraction of the pedons

**Plate 4:**
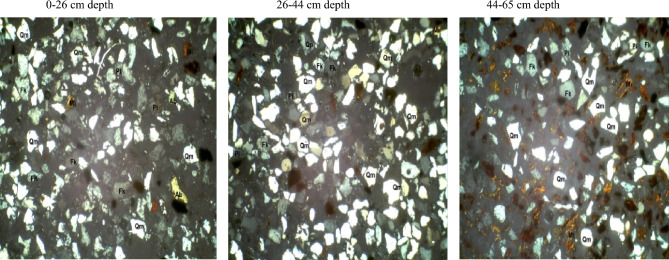
Petrographic images of fine sand fraction of pedon developed on basement complex rock. *Qm* monocrystalline quartz, *HM* heavy minerals, *Fk* potassium feldspar, *Rf* rock fragments, *Pl* plagiosclase feldpar.

In the fine sand fraction of soils developed over the basement complex rock (Table 4), quartz varied from 62% at 44–65 cm depth to 73% at the 26–44 cm depth. Plagioclase feldspar ranged from 9 to 26% in the 44–65 cm and 0–26 cm depth. Quartz/feldspar ratio and mica varied from 2.5 and 3% in the 0–26 cm to 6.9 and 25% in the 44–65 cm depth. Following the same trend, the mineral maturity index varied from 2.5 to 6.4% while there was no variation in the heavy minerals. The petrographic images of fine sand fraction of pedon developed on basement complex rock are showed in Plate 4.

**Table 4:** Mineralogical compositions of fine sand fraction of soils developed on different geological formations.

Geology	Depth (cm)	QZ	Fi	QZ/Fi	RF	Mica	Matrix	HM	Mmi
Basement complex	0–26	65	26	2.5	–	3	5	1	2.5
	26–44	73	16	4.6	–	6	4	1	4.6
	44–65	62	9	6.9	–	25	3	1	6.4
Coastal plain sand	0–40	80	11	7.3	2	2	4	1	6.1
	40–55	81	14	5.8	–	2	2	1	5.7
	55–82	72	23	3.1	–	–	3	2	3.2
Ewekoro formation	0–18	86	8	10.75	–	3	2	1	10.1
	18–38	80	12	6.7	–	1	6	1	6.7
	38–69	88	6	14.7	–	2	3	1	15.7

*QZ* quartz, *Fi* plagioclase feldspar, *QZ/Fi* quartz/feldspar ratio, *RF* total unstable rock fragment, *HM* heavy minerals, *MMI* mineral maturity index.

**Plate 5:**
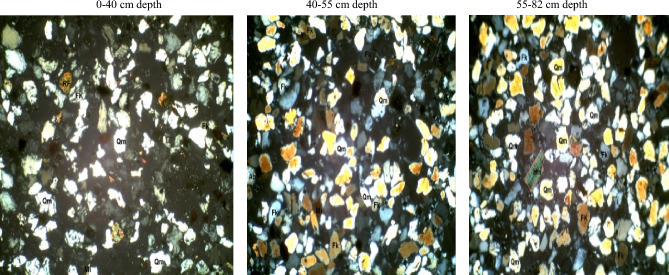
Petrographic images of fine sand fraction of pedon developed on Coastal plain sand. *Qm* monocrystalline quartz, *HM* heavy minerals, *Fk* potassium feldspar, *Rf* rock fragments, *Pl* plagiosclase feldpar.

Fine sand fraction from the coastal plain sand pedon has quartz varying from 72% at 55–82 cm depth to 81% at the 40–55 cm depth (B horizon). Plagioclase feldspar ranged from 11 to 23% in the 0–40 cm and 55–82 cm depth. Quartz/feldspar ratio varied between 3.1 and 7.3 in the 55–82 cm (C horizon) and 0–40 cm depth (Table 4). Mica which was not detectable in the C horizon was only 2% in the A and B horizons. There was no variation in the heavy minerals content except in the C horizon. The matrix varied between 2 and 4% while the mineral maturity index ranged from 3.2 to 6.1%. The petrographic images of fine sand fraction of pedon developed on coastal plain sand are showed in Plate 5.

**Plate 6:**
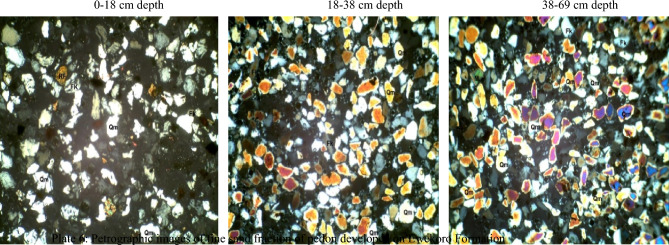
Petrographic images of fine sand fraction of pedon developed on Ewekoro formation. *Qm* monocrystalline quartz, *HM* heavy minerals, *Fk* potassium feldspar, *Rf* rock fragments, *Pl* plagiosclase feldpar.

The fine sand fraction from the Ewekoro formation pedon has quartz varying from 80% at 18–38 cm depth (B horizon) to 88% at the 38–69 cm depth (C horizon). Plagioclase feldspar ranged from 6 to 12% in the 38–69 cm and 18–38 cm depth. Quartz/feldspar ratio varied between 6.7 and 14.7% in the 18–38 cm (C horizon) and 38–69 cm depth (Table 4). Mica varied from 1% in the B horizon to 3% in the A horizon. There was no variation in the heavy minerals content along the horizon depth whereas **t**he matrix varied between 2 and 6%. The mineral maturity index ranged from 6.1 to 15.7% in the B and A horizon, respectively. The petrographic images of fine sand fraction of pedon developed on Ewekoro formation are showed in Plate 6.

Comparatively, Ewekoro formation had the highest quartz, quartz/feldspar ratio, matrix and the mineral maturity index followed by the soil from coastal plain sand and pedon from the basement complex rock recorded the lowest quartz and quartz/feldspar ratio, and the highest plagioclase feldspar contents. Pedons from the three geological formations had little to none of total unstable rock fragments. Ewekoro formation had the lowest mica content followed by those from coastal plain sand while the highest was found in pedon developed over the basement complex rock. There were little to no variations in the matrix and heavy mineral contents of the pedons from the three geological formations. The mineral maturity index was highest on the Ewekoro formation (> 19.5) while the least occurred in the basement complex rock (< 2.5) and coastal plain sand (< 3.2). In all the geological formations, the least mineral maturity index consistently occurred in the A horizons and the highest in the C horizons.

#### Coarse silt fraction of the pedons from the three different formations

**Plate 7:**
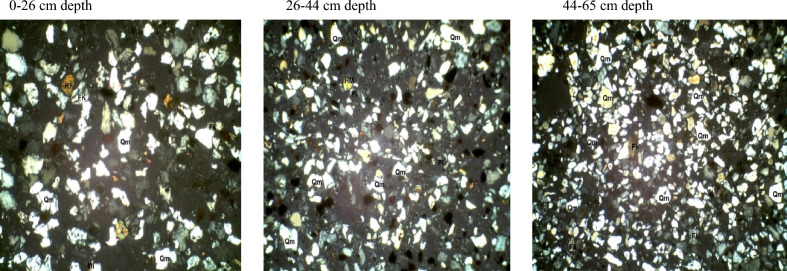
Petrographic images of coarse silt fraction of pedon developed on basement complex rock. *Qm* monocrystalline quartz, *HM* heavy minerals, *Fk* potassium feldspar, *Rf* rock fragments, *Pl* plagiosclase feldpar.

In the coarse silt fraction of soils developed over the basement complex rock (Table 5), quartz varied from 71% at 0–26 cm depth to 82% at the 26–44 cm depth. Plagioclase feldspar ranged from 2 to 21% in the 44–65 cm and 0–26 cm depth. Quartz/feldspar ratio and mica varied from 3.4 to 2% in the 0–26 cm to 40 and 10% in the 44–65 cm depth. Following the same trend, the matrix ranged from 4 to 6%. The mineral maturity index varied from 3.3 to 32% while the heavy minerals varied between 1 and 2%. The petrographic images of coarse silt fraction of pedon developed on Basement Complex rock are showed in Plate 7.

**Table 5:** Mineralogical compositions of coarse silt fraction of soils developed on different geological formations.

Geology	Depth (cm)	QZ	Fi	QZ/Fi	RF	Mica	Matrix	Hm	Mmi
Basement complex	0–26	71	21	3.4	–	2	4	2	3.3
	26–44	82	10	8.2	–	2	5	1	8.1
	44–65	80	2	40	1	10	6	1	32
Coastal plain sand	0–40	84	6	14	1	2	5	2	11.5
	40–55	82	12	6.8	–	1	2	3	6.7
	55–82	93	4	23.25	1	1	1	–	19
Ewekoro formation	0–18	90	5	18	–	1	2	2	19
	18–38	90	6	15	–	1	2	1	15.7
	38–69	90	4	22.5	–	1	4	1	24

*QZ* quartz, *Fi* plagioclase feldspar, *QZ/Fi* quartz/feldspar ratio, *RF* total unstable rock fragment, *HM* heavy minerals, *MMI* mineral maturity index.

**Plate 8:**
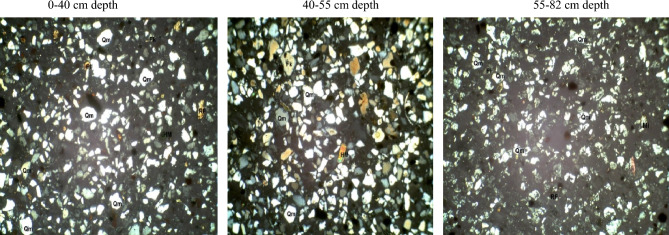
Petrographic images of coarse silt fraction of pedon developed on Coastal plain sand. *Qm* monocrystalline quartz, *HM* heavy minerals, *Fk* potassium feldspar, *Rf* rock fragments, *Pl* plagiosclase feldpar.

Coarse silt fraction from the coastal plain sand pedon has quartz varying from 82% in the B horizon to 93% in the C horizon. Plagioclase feldspar ranged from 4 to 12% in the C and B horizons respectively. Quartz/feldspar ratio varied between 6.8 and 23.25 in the B and C horizons respectively (Table 5). There was no variation in the unstable rock fragments and the mica content while the matrix varied between 1 and 5%. The heavy minerals were 2–3% in the A and B horizons while the mineral maturity index ranged from 6.7 to 19% in the B and C horizons, respectively. The petrographic images of coarse silt fraction of pedon developed on coastal plain sand are displayed in Plate 8.

**Plate 9:**
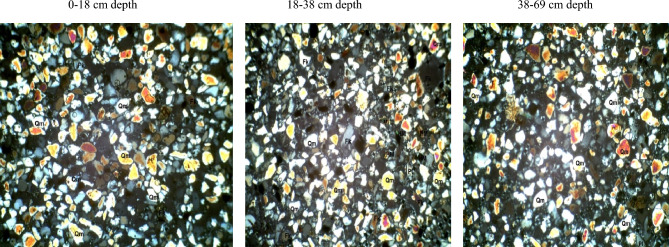
Petrographic images of coarse silt fraction of pedon developed on Ewekoro formation. *Qm* monocrystalline quartz, *HM* heavy minerals, *Fk* potassium feldspar, *Rf* rock fragments, *Pl* plagiosclase feldpar.

The quartz and mica contents of the coarse silt fraction of pedon in the Ewekoro formation were 90% and 1% though there were no variations in these minerals along the horizons (Table 5). The quartz/feldspar ratio varied from 18% in the A horizon to 22.5% in the C horizon. No unstable rock fragment was detectable while the mica content varied between 2 and 4%. The heavy minerals were 1–2% in the BC and A horizons while the mineral maturity index ranged from 15.7 to 24% in the B and C horizons, respectively. The petrographic images of coarse silt fraction of pedon developed on Ewekoro formation are showed in Plate 9.

## Discussion

The clay mineralogy of soils varies widely depending on such factors as the composition of the underlying rock, stage of soil formation, soil pH and soil moisture regime^18^. It is generally accepted that weathering intensity determines clay mineral types, quantity of pedogenic clay and the degree of profile differentiation. The clay mineralogy of soils of the basement complex or Upper Cretaceous Sandstones of Nigeria has been extensively studied^19^. There were similarities in the results obtained with the XRD and the SEM; while XRD identified the mineral compositions, SEM revealed the morphology and structural composition of the mineral components^20^.

The XRD patterns of clay fraction of soils developed on the basement complex rock, coastal plain sand and Ewekoro formation showed a great level of similarities. Mixed-layer illite, mica, kaolinite, quartz, hematite, anatase, goethite and chlorite were identified at varying degrees in the pedons developed on these geological formations. Kaolinite and quartz dominated the soil matrix. This can be adduced to the climate of the region which influences the mineral composition of the soils. The area is prone to high rainfall and leaching, and therefore soluble minerals tend to be washed away, leaving behind relatively resistant minerals like quartz and kaolinite. The coastal plain may have experienced leaching, leading to the accumulation of quartz and kaolinite in the soil matrix. Ajiboye et al.^21^ indicated kaolinite as the major clay type on soils developed over the basement complex in the well-drained Savanna ecology of Nigeria, whereas mica and other 2:1 sheet silicate was only present in trace quantity. The pedons developed on the basement complex rock was mineralogically more complex and diverse than those of the other geological formations.

In horizon A kaolinite occurred at moderate level, this could be that the transition from the A horizon (topsoil) to the C horizon (parent material or unweathered material) in soils formed on Basement complex rocks were associated with changes in weathering conditions and soil development. Several factors can contribute to the increasing quantities of kaolinite from the A horizon to the C horizon in X-ray diffraction (XRD) analyses, including weathering processes whereby the A horizon being typically more weathered than the C horizon due to exposure to environmental conditions over time^22^, chemical weathering processes, such as hydrolysis and leaching, can result in the breakdown of primary minerals in the rock into secondary minerals like kaolinite^23^. Kaolinite is a product of the weathering of feldspar minerals commonly found in Basement complex rocks. Also, the A horizon is often subject to leaching, where water percolates through the soil, dissolving and transporting minerals downward. The leached materials may accumulate in lower horizons, leading to the enrichment of kaolinite in the C horizon through a process known as illuviation^24^.

This difference in parent material contributes to the variation in this mineralogical complexity. Basement complex rocks are typically older and more complex in terms of their mineral composition compared to the sediments found in coastal plains. Basement complex rocks often consist of a wide range of minerals, including igneous, metamorphic, and sedimentary rocks that have undergone extensive geological processes over millions of years. In contrast, coastal plain sediments are usually younger and primarily composed of weathered and transported materials from nearby sources, such as rivers and eroded mountains. Furthermore, Basement complex rocks are usually formed through tectonic processes like volcanic activity, mountain building, and metamorphism, which can introduce a diverse range of minerals into the rock. On the other hand, coastal plains are often formed through sedimentation and erosion processes, which tend to sort and transport sediments, resulting in a more limited range of mineral types. Pedon 2 and 3 of the basement complex rock were the richest in terms of mineralogical compositions and had greater proportion of illite/mica than other pedons. It’s possible that Pedons 2 and 3 originated from a parent rock with a higher content of minerals like illite/mica compared to the other pedons. This could be due to variations in the sedimentary or igneous protoliths that formed the basement complex rock.

The coastal plain had the highest quantity of hematite while the least was recorded in the Ewekoro formation, the coastal plain, being a depositional area, could have accumulates sediments that include hematite derived from eroded rocks in the surrounding regions. The Pedons from Ewekoro formation were the poorest in the 2:1 mineral composition as demonstrated in their low quantity of illite/mica and high quartz contents. The effect of this mineralogical compositions is reflected in the poor soil chemical properties of the Ewekoro formation (Table 6). In intensely weathered soils such as oxisols, kaolinite and halloysite are the predominant clay types; other minerals such as gibbsite and sesquioxides are also present^25^. In the B horizon of pedons from the basement complex rock, illite/mica was abundant while quartz occurred at moderate level while in those of coastal plain sand, quartz and illite occurred at moderate levels whereas kaolinite and quartz were abundant in the B horizons of pedons on the Ewekoro formation. The high quantity of illite/mica in basement complex formation was due to the fact that these rocks typically contain minerals like feldspar, mica, and quartz. Illite and mica are common clay minerals found in these rocks, and their weathering and decomposition contribute to the abundance of illite/mica in the B horizon.

**Table 6:** Comparative analysis of soil chemical properties of the three geological formations.

Locations	pH	SOC (%)	TN (%)	P (mg kg^−1^)	K	Ca^2+^	Mg (cmol kg^−1^)	Na	H^+^	CEC
Coastal plain sand	6.02 a	3.74a	0.24b	7.12a	0.28ab	1.69c	1.56a	0.73a	0.11a	4.37a
Ewekoro formation	5.78 a	3.35ab	0.22c	6.84b	0.27c	1.73b	1.52a	0.68a	0.11a	4.31ab
Basement complex	5.85a	3.86a	0.25a	7.37a	0.29a	1.82a	1.56a	0.74a	0.11a	4.52a

*Soc* soil organic carbon, *TN* total nitrogen, *P* phosphorus, *K* potassium, *Ca* calcium, *Mg* magnesium, *Na* sodium, *Cec* cation.

In terms of mineralogical richness, the geological formations could be ranked as: basement complex rock > coastal plain sand > Ewekoro formation. According to Abbas et al.^26^ soil mineralogy has a profound influence on the chemical characteristics and dynamic behaviour of soils and the environment. Ewekoro has the least chemical properties due to high weathering and leaching. Weathering typically results in the release of nutrients from rocks into the soil. However, the rate and extent of nutrient release can vary. The weathering in the Ewekoro formation might have been extensive, leading to a higher concentration of certain nutrients in the soil which might have been loss through leaching^27^. Again, highly weathered soils may also be more susceptible to erosion because the finer particles resulting from weathering are often more easily transported by wind or water^28^. This could have implications for soil fertility and stability.

Furthermore, scanning electron microscopy (SEM) is one of the most versatile instruments available for the examination and analysis of the microstructural characteristics of solid objects such as soil^29^. The elemental compositions of clay fraction of soils developed over the three geological formations as revealed by the SEM were carbon, oxygen, silicon, aluminum and iron at varying amounts. This was a reflection of the composition of the parent materials and the weathering processes that have acted upon them over time. The abundance of silicon, oxygen, and carbon in all the basement complex Ewekoro formations show that that these elements are integral to soil mineralogy, structure, fertility, and the overall functionality of the soil ecosystem. Understanding the interplay of these elements helps in managing and optimizing soil health for sustainable agriculture and ecosystem services. For example, the combination of silicon, oxygen, and carbon influences the overall fertility of the soil. Silicon contributes to mineral stability and plant health, while oxygen and carbon are crucial for the microbial activity and nutrient cycling that support plant growth^30^. Silicon-rich minerals, such as quartz, contribute to soil texture. The arrangement of mineral particles affects soil structure, permeability, and drainage. Organic matter derived from carbon contributes to soil aggregation and structure. Most importantly, carbon is a central component in the organic matter that undergoes decomposition by soil microorganisms^31^. This decomposition process releases nutrients, making them available for plant uptake. The shapes of the mineral crystals in the coastal plain sand and ewekoro formation, showed that they have undergone severe weathering^32^ and that the pedons from these geological formations are therefore at advanced stages of soil weathering.

The mineralogy trends reflected different parent materials but suggested that much of the soil mineral was dependent on weathering. Quartz was the most dominant mineral species scattered across the slides. They occurred mostly as mono-crystalline crystals. It appears in thin sections as relatively clear colourless grains having a weak birefringence and a low refractive index that is only slightly higher than that of the mounting medium. Most of the monocrystalline quartz crystals show angular to subrounded shapes, slightly undulose to non-undulose extinctions. Many of the quartz grains were not morphologically enlongated or flattened and do not show parallel crystallographic orientation. This is an indication that the sediments were probably sources from acid igneous rocks with minor input from metamorphic sources^33^. Similar results were obtained by Nton and Adeyemi^34^ while studying the petrography, compositional characteristics and stable isotope geochemistry of the Ewekoro formation.

Like quartz, feldspars in thin sections are typically clear, colourless and show low birefringence, but they can be distinguished from quartz by their cleavage, twining and refractive indices. Nevertheless, distinguishing between untwinned orthoclase and quartz can be difficult. However, feldspar grains may sometimes be partly decomposed, and then they appear cloudy or turbid in contrast to quartz grains, which are invariable unaltered and relatively clear. In the studied sandstones, potash feldspar (Fk) is much more prevalent than plagioclase (Pl) among the various types of feldspar. There are two reasons for this: K-feldspar has a greater chemical stability than plagioclase^35^, and so the latter is altered preferentially in the source area. In addition, K-feldspar is much more common in continental basement rocks (acid gneisses)^35^, which are probably the provenance of the studied samples.

Micas were identified by their platy shape and parallel extinction. Biotite is the most abundant mica and had a brown–green pleochroism which masks the interference colours. Biotite was observed in low abundance (1–6%) in some samples while observed in rather high abundance (10–25%) in others. Biotite occurs as large detrital flakes which are concentrated along partings, laminae and bedding planes. This distribution is primarily a function of sorting and is determined largely by the hydraulic behavior of the mica flakes^35^.

Matrix is seen as brownish patches on the slides and this is as a result of intensive weathering and ferruginisation of the sediments during the course of transportation. These are dominantly fine quartz crystals and clay minerals. Rock Fragments—these minerals are least seen on the slides because most of them have been weathered away. They appear in high relief as rough crystals occurring as low-grade sedimentary lithics as well as metamorphic lithics.

A “pedon” refers to the basic unit of soil classification, and it is a three-dimensional soil body that is used to describe and study soil properties and horizons^36^. It typically extends from the soil surface down to the lower limit of plant roots or to a depth of about 1.5 m. The study of pedons helps soil scientists understand soil formation, classification, and its various characteristics. Therefore, in coastal plain sand soils, the uppermost layer is the O Horizon which is a relatively thin coastal sand due to the fast decomposition of organic matter in the warm and humid tropical climate. Underlying this is the A Horizon which is sandy in texture, reflecting the characteristics of coastal plain sand soils. Overall, the pedon is characterized by a soil profile adapted to the specific environmental conditions, including sandy textures, leaching processes, and the influence of the tropical climate on organic matter decomposition^37^.

In basement complex rocks, the soil is derived from the weathering of basement complex rocks such as granite, gneiss, and schist. These rocks are often hard and crystalline, contributing to the mineral composition of the soil. The structure exhibits blocky or granular patterns, while crystalline structures from the original rock were still visible in some areas. The pedon in basement complex rock soils is characterized by its origin from crystalline rocks, distinctive colorations, and textures influenced by weathering, as well as the development of soil horizons and drainage patterns typical of tropical environments^38^.

The Ewekoro Formation typically consists of sedimentary rocks, including limestone and is known for its characteristic soils that have developed over time and the soils formed on it exhibit specific properties based on the parent material and environmental factors^39^. The soils formed on limestone are relatively fine-textured, with a mix of clay, silt, and fine sand. The soil profile consists of distinct horizons, including an A horizon (topsoil), B horizon (subsoil), and a C horizon (parent material). The development of these horizons is influenced by the weathering of the underlying limestone and the accumulation of organic material. Soils formed on the Ewekoro Formation are influenced by the alkaline nature of limestone. Consequently, the soils exhibit a relatively high pH, which can impact nutrient availability. Agricultural practices on soils of this formation may need to account for the alkaline nature of the soil. Liming might be necessary to adjust pH levels, and nutrient management strategies may be implemented to optimize crop growth.

According to Nwajide and Reijers^40^ classification of minerals using the mineral maturity index, profiles developed over the basement complex rock and the coastal plain sand could be regarded as sub-matured and this could have contributed significantly to the native fertility of these soils. The relatively low quartz and quartz/feldspar ratio, and high mica content of these profiles, further supported this assertion. The presence of a noticeable quantity of mica in these soils suggests the ability of the soils to supply native potassium^41^. Mica contains potassium within its mineral structure, and over time, weathering processes can release potassium into the soil. As mica weathers, it breaks down into smaller particles, and the potassium it contains becomes available for plant uptake. This indicates that the profiles are relatively rich in weatherable minerals and could therefore support low input agricultural system of a typical smallholder farm in Nigeria where the chemical K fertilizer is scarce and expensive^42^. Hence, due to the limited utilization of fertilizers in Nigerian crop production, a reliance on the intrinsic reserves of soil nutrients is necessary. Mineralogy stands out as a crucial component in this context, as highlighted by Vitousek et al.^43^, Vanlauwe et al.^44^, and Keskinen et al.^45^. Profiles from the Ewekoro formation were at super-matured state as symbolized in their high quartz, quartz/feldspar ratios and mineral maturity index, and low mica content. This implied that there were no more weatherable minerals in the profiles from Ewekoro formation. Soils with high quartz content often have poor nutrient-holding capacity because quartz is an inert mineral^46^ meaning it does not contribute significant nutrients to the soil. Soils dominated by quartz may be well-drained but can lack essential nutrients for plant growth. High quartz content alone doesn’t contribute to soil fertility, and additional amendments or organic matter may be needed to enhance nutrient availability. Therefore, large quantities of organic manure are needed to enhance nutrient availability. High quartz/feldspar ratios may indicate a more mature soil with extensive weathering^47^. Highly weathered soils might have leached away some nutrients, leading to lower fertility^48^. To sustain crop production, Organic Matter Addition, cover cropping, mulching, targeted fertilizer application based on soil testing, agroforestry, crop rotation, among others are necessary. The mineral maturity index is a measure of the degree of weathering in a soil. A high mineral maturity index indicates well-developed weathering, while a low index suggests less weathering. More mature soils may have experienced greater mineral breakdown, potentially leading to nutrient depletion. However, the relationship between mineral maturity and fertility is complex and influenced by various factors. Mica is a source of potassium, and soils with low mica content may have lower potassium availability. Low potassium availability can negatively affect plant growth, as potassium is an essential nutrient for various physiological processes. In such cases, supplemental potassium fertilization might be necessary to meet plant requirements.

## Conclusion

This study provides an integrative examination of the mineral compositions and diversities of soils developed over the basement complex rock, coastal plain sand and the Ewekoro formation. Using X-ray diffractometry (XRD) and Scanning Electron Microscopy (SEM), the mineral contents, elemental compositions and the morphologies of the clay, coarse silt and fine sand fractions of the soils from these geologies at different topographic positions were identified, described and compared.

There were similarities in the results obtained with the XRD and the SEM; while XRD identified the mineral compositions, SEM revealed the morphology and structural composition of the mineral components. Mixed-layer illite, mica, kaolinite, quartz, hematite, anatase, goethite and chlorite were identified at varying degrees in the pedons developed on these geological formations though kaolinite and quartz dominated the soil matrix. The most abundant elements in the basement complex and Ewekoro pedon were oxygen, carbon and silicon whereas in the coastal plain sand pedon, oxygen, carbon and aluminum were the most abundant element. The order of mineralogical complexity of the pedons based on the geologies were: basement complex > coastal plain sand > Ewekoro formation. Thus, profiles developed on Ewekoro formation were the most weathered as symbolized in their chemical properties and mineralogical compositions.

The petrographic evaluation of the mineralogical diversity of the fine sand and coarse silt fractions of pedons from the three geological formations revealed that all the pedons were quartz-rich and with varying degrees of mineral complexity and maturation. The overlap and distinctness among the geologies was apparent of their different weathering stages. Petrographic analysis also indicated profile from Ewekoro formation to be the most weathered as earlier indicated in the chemical properties, XRD and SEM analyses. There were relatively few weatherable minerals left in the underlying rocks on the profiles of soil from Ewekoro; based on the mineralogical complexity and maturities of most of the C horizons of the profiles. The use of such soils for agriculture would therefore require huge agro-inputs investments such as improved seeds, modern and adapted cropping systems coupled with sound principles of soil management through the use of integrated soil fertility management.

## Materials and methods

### Description of the study area

This research focused on three geological formations: Basement complex rocks, Coastal Plain Sand, and Ewekoro Formation. The study sites were specifically chosen as Lanlate, Akute, and Ewekoro to represent each of these formations, respectively.

Lanlate (Fig. 1) is situated within the latitude range of 7° 350ʹ N to 7° 450ʹ N and the longitude of 30° 400ʹ E. It is geographically bounded by the Oke-Ogun area of Oyo State to the north, and by Ogun State in the west and south. Lanlate falls within the tropical hinterland climatic belt due to its latitude. The region experiences a double peak in rainfall, particularly in the southern part adjacent to Ibadan in Oyo State and Ogun State. The annual rainfall in this area ranges between 1500 and 2000 mm. The relative humidity is high, exceeding 80% in the morning and decreasing to 50–70% in the afternoon. The mean annual temperature ranges around 80 °C. Despite prolonged human interference with the original forest vegetation, Lanlate has managed to preserve its forest vegetation species. Trees are more abundant than grasses in this area. The forest reserve in Lanlate represents a significant remaining portion of the original vegetation cover in the region.

Akute, located in the Ifo Local Government of Ogun State, Nigeria, serves as a significant boundary between Lagos and Ogun State. Its precise geographical coordinates are 6° 40ʹ 56ʹʹ North, 3° 22ʹ 34ʹʹ East, and it was originally named Akute-Oja. This area experiences a substantial amount of rainfall, with annual precipitation surpassing 2000 mm. Geologically, Akute is characterized by sedimentary rocks that harbor the preserved records of organic life spanning various epochs. The soils in this region, being derived from sediments, play a crucial role in supporting plant growth and possess notable economic significance^49^.

Ewekoro is located in Ogun State, Nigeria, and is surrounded by various neighboring regions. To the west, it is bounded by Benin Republic, while Lagos State lies to the south. In the north, Ewekoro is bordered by Oyo and Osun States, and in the east, it is adjacent to Ondo State. Notably, Ewekoro is renowned for being the site of the West African Portland cement quarry.

Geographically, Ewekoro is situated between longitude 3° 05ʹ E to 3° 15ʹ E and latitudes 6° 40ʹ N to 6° 55ʹ N. The town is situated within the tropical rainforest of the subequatorial southwestern region of Nigeria. As a result, it experiences distinct wet and dry seasons, which are characteristic of the country’s climate. The average relative humidity in the area ranges between 75 and 95%. Additionally, the mean annual rainfall is approximately 1500–2000 mm, and the mean monthly temperature ranges from 22 to 22.5 °C^50^.

### Geology of the area

Ewekoro is situated in the sedimentary region of southwestern Nigeria, specifically belonging to the tertiary-formed Palaeocene and Eocene periods. The majority of the area is a potential artesian basin, allowing for the extraction of groundwater. Reyment^50^ provided an overview of the Albran and younger Palaeographic history of Nigeria, highlighting the transgressive and regressive phases, as well as the characteristics of the sediment. The geology of Ogun State consists of both sedimentary and basement complex rocks that form the foundation of the state’s surface. Within this geology, there are intercalations of argillaceous sediment. The rocks in this region are generally soft and crumbly, but in some locations, they are cemented by ferruginous and siliceous materials. The sedimentary rock sequence of Ogun State includes the Abeokuta Formation, which directly overlays the basement complex. The basement complex rocks of Nigeria comprise a collection of crystalline igneous and metamorphic rocks dating from the Precambrian to the lower Proterozoic era. These rocks can be broadly classified into three lithological units: the migmatite–gneiss–quartzite complex, the schist belt, and the older granites^7^. In the southwestern part of the country, Rahaman^51^ categorized the basement complex into distinct groups, including the migmatite-gneiss complex, slightly migmatized to unmigmatized paraschist and metaigneous rocks, charnokitic rocks, older granites, and unmetamorphosed doloritic dykes. This is further overlaid by the Ewekoro, Oshosun, and Ilaro Formations, and ultimately covered by the coastal plain sands of the Benin formation. The Benin formation is characterized by alternating sequences of limestone and shale^52^.

Limestone is a type of sedimentary rock primarily composed of calcite (CaCO_3_). It forms through organic or inorganic processes^53^. Marble, on the other hand, is a metamorphic rock with a crystalline texture that primarily consists of calcite (CaCO_3_), dolomite [CaMg(CO_3_)_2_], or a combination of both. Marble is the result of the metamorphosis of limestone. Both limestone and marble are carbonate rocks dominated by the mineral calcite (CaCO_3_).

### Field work

A total of nine profile pits of 2 m × 1 m × 2 m size were dug in all the three locations with three located in Lanlate, three in Akute, and three in Ewekoro for the purpose of this study.

To prepare the soil samples, they were first air dried and then crushed before being passed through a 2 mm sieve mesh to ensure uniform particle size. This sieving process allowed for the determination of the percentage composition of sand, silt, and clay in the soil, enabling the segregation of minerals and facilitating quantitative analysis.

For the analysis of particle size distribution, the dispersed soil samples were subjected to further treatment. Clay particles were allowed to settle after 2 h of dispersion, and the sediments were flocculated and collected. The clay slurry was then shaken again and decanted after 1 min. The remaining sediment was identified as the sand fraction, which was subsequently washed multiple times to eliminate any remaining clay particles. The sand sediment fraction was dried and divided into fine and coarse fractions using suitable sieves. The weight of each fraction was recorded.

The fine sand particles were processed to remove any iron oxides present and subsequently dried. These fine sand fractions were then ready for petrographic analysis. In addition, a small amount (approximately 10 g) of each soil sample was finely ground and preserved for the determination of organic carbon and total nitrogen content.

### Laboratory procedure for X-ray diffractometry (XRD)

The soil samples obtained from each profile horizon were subjected to a series of preparation steps. Firstly, the samples were air dried and sieved through a 2 mm mesh to ensure uniformity. To eliminate any organic materials that could bind the soil particles together, the samples were treated with a 50% hydrogen peroxide solution. Next, each soil sample was dispersed in a diluted solution of sodium hexametaphosphate (Calgon) to serve as a dispersant. The dispersion process involved transferring the soil sample into a designated cup and stirring it mechanically for approximately 15 min.

Following the dispersion step, 500 ml of water (H_2_O) was added to the soil and vigorously shaken. The mixture was then allowed to stand for approximately 2 h, after which the supernatant was carefully decanted. The resulting clay suspension was then transferred to a properly labeled container. To facilitate flocculation of the concentrated clay sample, approximately 20 ml of concentrated sodium chloride (NaCl) solution was added. To remove any iron oxide present in the clay samples, the DCB (Diothionite Citrate Bicarbonate) method, as described by Jackson et al.^42^, was employed. From each of the 30 flocculated clay samples, around 2 ml of the clay slurry was collected in a well-labeled centrifuge tube. Subsequently, 6 ml of a 1 M solution of sodium citrate trihydrate and 3 ml of concentrated sodium bicarbonate solution were added to the tube and thoroughly mixed. The mixture was then placed in a water bath heated to 76 °C.

After a brief period of heating, 0.5 g of sodium diothionite powder was introduced to the clay suspension in the centrifuge tube. The tube was left undisturbed in the water bath for approximately 50 min. Regular stirring of the mixture occurred at intervals of 10 min throughout this duration.

After undergoing heating, the clay solution underwent centrifugation at 2500 rpm for 15 min, and the resulting supernatant was subsequently discarded. Following that, in each of the 30 samples, 10 ml of 1 M sodium acetate (pH 7) was added and thoroughly mixed. The mixture was then centrifuged at 1500 rpm for 15 min, and the supernatant was once again discarded. This entire process was repeated, and subsequently, all the samples were divided into two equal portions of 15 each. One portion was designated for magnesium saturation, while the other portion was allocated for potassium saturation.

### Magnesium saturation

For the magnesium saturation process, 10 ml of magnesium acetate (pH 7) was added and mixed with each of the fifteen magnesium saturation samples. The mixture was then allowed to sit for an hour before undergoing centrifugation, and the resulting supernatant was discarded. Subsequently, another 10 ml of magnesium chloride was added, mixed thoroughly, and centrifuged at 1500 rpm. Once again, the supernatant was discarded.

This procedure was repeated, and to the resulting mixture, 1 ml of 50% ethanol was added. A drop of glycerol was then mixed using a vortex genie mixer. The clay dispersion was then quantitatively transferred into a ceramic crucible and dried at room temperature. The dried clay samples were subsequently quantitatively transferred into a ceramic mortar and milled to achieve homogeneity. Finally, the powdered clay samples were individually packaged in appendoph tubes and shipped to Ktgeoservices Inc. in Gunnison, California, USA, for XRD analysis.

### Potassium saturation

Potassium Saturation is a confirmatory test to distinguish between the presence of Kaolinite and Chlorite.10 ml of potassium accetate (pH 7) was added and mixed with all the fifteen samples for the process and left for an hour, centrifuge and supernatant was discarded. 10 ml of potassium chloride was added too. Mixed and then centrifuge at 1500 rpm and the supernatant was also discarded. This procedure was repeated and 10 ml of methanol was added and stirred vigorously, after which it was centrifuge at 1500 rpm and the supernatant was discarded. This procedure was also repeated and 1 ml of 50% ethanol was added followed by a drop of glycerol which was mixed on a vortex genie mixer. The clay samples were transferred into labeled crucibles and incubated for 12 h at 550 °C in a muffle furnace. The incubated clay samples were quantitatively transferred into a ceramic mortar and milled to homogenize the clay. This powdered clay samples were package each in an appendoph tube and shipped for XRD analysis at Ktgeoservices Inc., Gunnison, California, USA.

### Removal of iron oxide

To prevent the interference of minerals, the natural coating of iron oxide on sand grains from the field needs to be eliminated. The method employed to remove the free iron oxide is the Dithionite-citrate Bicarbonate (DCB) method developed by Jackson^54^. In this method, molar solutions of sodium bicarbonate and sodium citrate are utilized.

To begin, 0.15 ml of sodium citrate is introduced into sample bottles containing the fine sand fraction of each soil sample. The bottles are then placed in a warm water bath set at a temperature below 70 °C and left undisturbed for a duration of 15 min. After this initial step, approximately 30 ml of sodium bicarbonate is added to the mixture. The contents are thoroughly stirred for several minutes. Next, the bottles are removed from the water bath, and the supernatant (liquid portion above the sediment) is carefully decanted or poured off, leaving behind the sand grains with reduced iron oxide coating. By following this procedure, the iron oxide coating on the sand grains can be effectively removed using the DCB method.

### Petrographic analysis

The geology laboratory at the University of Ibadan conducted a comprehensive petrographic analysis. Petrography entails the examination of rocks and minerals using a microscope. This technique involves the preparation of cross sections, which aid in the identification of rocks, minerals, and ores, as well as the characterization of various properties like cleavage, twinning, and reflectance. For petrographic analysis, two types of specimens are typically prepared: thin sections and polished bulk specimens.

Polished bulk specimens resemble metallographic samples, as their surfaces are prepared for examination under a reflected light microscope. Conversely, thin sections are observed using a transmitted polarized light microscope. The general process for creating transparent thin sections involves several steps: sectioning, vacuum impregnation, grinding, cementing to a slide, resectioning, and final grinding and polishing. The preparation of thin sections is notably more challenging compared to polished bulk specimens.

In general, a thin section is meticulously prepared to achieve a thickness of approximately 30 µm, ensuring near-perfect parallelism throughout the section.

### Preparation procedure

The process of creating thin sections began with bulk sectioning to obtain a section approximately 3 mm thick. For less consolidated materials, a thicker section, up to 10 mm in thickness, was obtained. The next step involved thorough cleaning and vacuum impregnation with EpoThin Low Viscosity Epoxy to fill the pores and provide mechanical support to the specimen material.

Subsequently, the specimen “chip” was ground to achieve a flat and smooth surface, ensuring the absence of any significant deformations. Depending on the nature of the materials, the chip was categorized into either Soft Materials (such as Sulfides, Carbonates, Sandstones, etc.) or Hard Materials (including Granite, Basalts, Quartz, Chert, and Ores) when selecting appropriate grinding and polishing abrasives.

To determine if the entire surface of the chip had been properly ground, the ground surface was held against light at approximately a 45-degree angle. An evenly reflective surface indicated proper grinding, while a non-uniform and dull surface suggested the need for additional grinding.

The next step involved cementing the chip to a glass slide. Prior to this, the specimen was thoroughly cleaned to remove any loose abrasive or residues and dried. One side of the glass slide was pre-ground to establish uniform thickness and provide a roughened surface for better bonding. The thickness of the slide was controlled using the 30-8001 Glass Slide Holder or the Petro Thin system. Loose silicon carbide abrasive powders with grit sizes of 600 or 1000 (P1200-P2000) were typically used on a cast iron lap for grinding the slides.

To attach the chip to the slide, epoxy was used according to the recommended instructions. The ingredients were blended thoroughly but gently to prevent excessive air bubble formation. After allowing the mixture to sit for a few minutes, the epoxy was sparingly applied to the ground surfaces of the chip and the slide. The chip, adhesive side down, was carefully placed onto the coated slide, and moderate pressure was applied to remove excess adhesive and air bubbles by moving the chip back and forth over the slide. For achieving a uniform adhesive thickness, the use of the Petro bonding fixture was recommended. The fixture was placed on a hot plate to expedite epoxy curing, ensuring the temperature did not exceed 50 °C (122 °F). Applying heat shortened the curing time to approximately two to three hours, whereas without heat, the thin sections would require 40–48 h to cure properly, depending on the ambient temperature.

Finally, re-sectioning of the specimen was performed to reduce the chip thickness and minimize grinding time. This re-sectioning process could be accomplished using an IsoMet Precision Saw or the Petro Thin Sectioning System.

### Statistical analysis

Data was subjected to Analysis of variance (ANOVA) using GenStat 12th edition. Significant means were separated using the Duncan’s Multiple Range Test at 5% probability.

### Ethical approval

The author confirm that all the research meets ethical guidelines and adheres to the legal requirements of the study country.

### Compliance with international, national and/or institutional guidelines

Experimental research (either cultivated or wild), comply with relevant institutional, national, and international guidelines and legislation. Experimental studies were carried out in accordance with relevant institutional, national or international guidelines or regulation.

## Data Availability

All datasets generated and/or analysed during the current study are included in this article.
